# Growth and Development of Stevia Cuttings During Propagation with Hormones in Different Months of the Year

**DOI:** 10.3390/plants9030294

**Published:** 2020-03-01

**Authors:** Ma Claudia Castañeda-Saucedo, Ernesto Tapia-Campos, Jessica del Pilar Ramírez-Anaya, Jaqueline Beltrán

**Affiliations:** 1 Laboratory of plant physiology, Centro Universitario del Sur (Cusur), Universidad de Guadalajara (UDG), Av. Enrique Arreola Silva 883, Centro, C.P. 49000 Cd. Guzmán, Jalisco, Mexico; jessica@cusur.udg.mx (J.d.P.R.-A.); yaqelinbl@hotmail.com (J.B.); 2 Centro de Investigación y Asistencia en Tecnología y diseño del Estado de Jalisco (CIATEJ), Av Normalistas 800, Colinas de La Normal, Guadalajara, 56230 Jalisco, Mexico

**Keywords:** indol-3 butyric acid, alfanaphthylacetamide, asexual propagation, cuttings

## Abstract

Stevia is an important non-caloric sweetener that has health-beneficial properties. The objective is to evaluate growth, development, and rooting of stevia plants during different seasons of the year using growth hormones. Eight experiments were set up in Ciudad Guzman, Jalisco, Mexico, with three treatments (T): T1, indol-3 butyric acid (IBA) 7.4 mM; T2, alphanaphthylacetamide (ANA) 6.4 mM + IBA 0.3 mM; and T3, control. The variables evaluated were rooted plantlets, plant height, root length, number of leaves, stem diameter, leaf dry weight, stem dry weight, root dry weight, leaf area, shoot biomass, total biomass, as well as development and growth indexes. Four samplings were conducted in each experiment. The results show that the most appropriate months for propagating stevia cuttings are February, March, April, May, and July, when 96% to 99% of the cuttings rooted. The hormones had the best results related to production of root development. The control was outstanding only in variables related to production of shoot biomass and not to root development. It is concluded that stevia can be propagated vegetatively using cuttings treated with IBA 7.4 mM or ANA 6.4 mM + IBA 0.3 mM, preferable in the period from February to July, with the exception of June.

## 1. Introduction

Stevia (*Stevia rebaudiana* Bertoni), which originated in Paraguay [1], belongs to the Asteracea family, native to the South American tropics. It adapts easily to subtropical and tropical regions that have ideal conditions for its growth and development [2]. Stevia is produced commercially in Brazil, Paraguay, Central America, Thailand, Korea, and China [3]. Worldwide, 32,000 ha are cultivated with stevia; of this area, China cultivates 75% [4]. Japan uses large quantities of stevia; the total market value of stevia sweetener is estimated to be around 2–3 billion yen/year [5]. It is considered a novel profitable crop, with promise in México [2]. Likewise, other countries such as Sri Lanka consider stevia an alternative to meet the high demand for sugar in the pharmaceutical, confectionary, and soft drink industries [6]. In India, farmers have begun to produce stevia for the diabetes market [4]. Its principal active compounds are steviosides, rebaudioside, and steviolbioside [7], which, isolated, can be up to 300 times sweeter than sucrose. It is the best substitute for cane sugar because of its natural origin and its low caloric content [8]. Moreover, stevia’s antioxidant effect has been shown to reduce oxidative stress [9]. Because of this and other therapeutic applications [2], with beneficial effects on type II diabetes and obesity [8] and antibacterial and anticancer effects [2], stevia is considered a medicinal plant [10]. The worldwide concern for chronic degenerative diseases and the demand for healthy foods in western societies have stimulated a strong interest in stevia as an alternative to sucrose and artificial sweeteners [11]. The natural antioxidants in stevia can replace synthetic antioxidants such as BHA (butylated hydroxy anisole) and BHT (butylated hydroxytoluene), which have been restricted in their use because they are potential carcinogens [2].

In the next few years, we hope that stevia production will meet, or surpass, the demand. To this end, finding alternative areas of stevia production to obtain leaves and extracts is important [11]. Sexual propagation is limited [12], given that stevia is a self-incompatible plant whose pollination is entomophilous [10], which generates a high degree of heterogeneity in the plants produced. Moreover, the seeds of this species are small and the germination percentage is low, 25.51%–40% [13]. For this reason, the most used propagation system is vegetative. Mother plants are used to produce cuttings, which are planted both in beds and in vitro. Vegetative propagation is the most common and effective alternative, producing uniform vigorous plants in a short time [14]. The use of hormones is controversial. Some authors have reported that IBA produces better rooting in young cuttings of *Myrceugenia exsucca* [15], in *Ficus binnendijkii* [16] and stevia [14]. However, others have reported that hormones do not promote more rooting in stevia [17]. There have also been reports that the month or time of propagation varies depending on the genotype and climatic conditions [13,16,18]. Thus, it is important to study the effect of hormones on plantlet rooting and the best month in which to propagate stevia. The objective of this study is to determine the best propagation time for stevia cuttings and to compare two root-promoting hormones.

## 2. Results

### 2.1. Temperatures during the Development of the Experiment

The average daily low temperature recorded during the experiments oscillated between 5.8 °C in January to 17.35 °C in August. The average daily high temperature varied between 32 °C in January to 42.7 °C in May, and the mean temperature was 16.4 °C in January and 24.9 °C in May (Table 1). November and February had the lowest average temperatures, while May had the highest average temperature (24.9 °C). From April through June, daily high temperatures were above 40 °C.

### 2.2. Stevia Plantlet Growth and Development 28, 35, 42, and 49 Days after Establishment (Dae)

**Figure 1:**
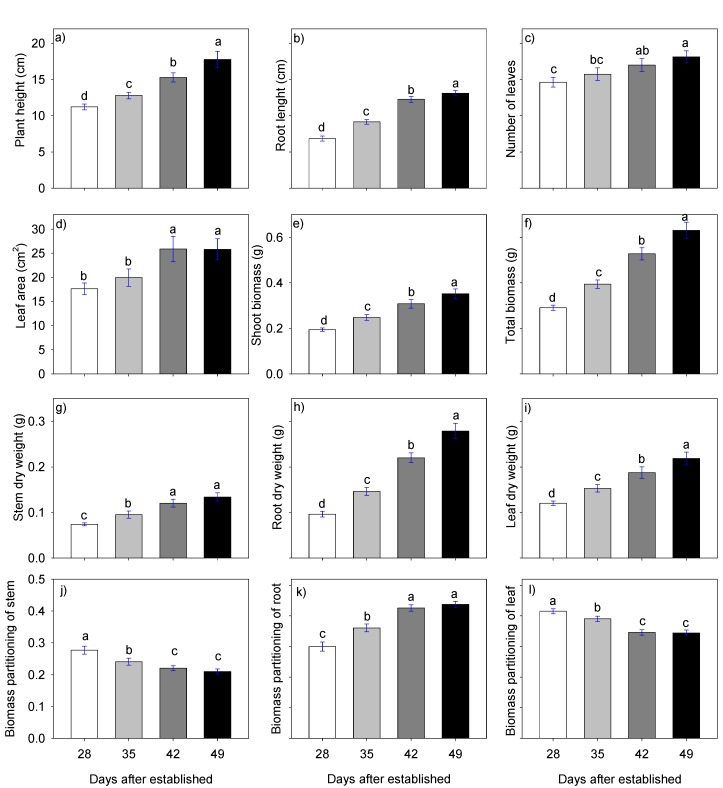
(**a**) Plant height, (**b**) root length, (**c**) number of leaves, (**d**) leaf area, (**e**) shoot biomass and (**f**) total biomass, (**g**) stem dry weight, (**h**) root dry weight, (**i**) leaf dry weight, (**j**,**k**,**l**) biomass partition of stem, root, and leaf of stevia plantlets 28, 35, 42, and 49 days after establishment. Each value represents the average of 120 data (5 replications of each of the three treatments of each of the 8 experiments). Means with the same letter in each figure are not significantly different (Tukey, *p* < 0.05).

On days 28, 35, 42, and 49 after establishing the experiments, significant differences were found for the variables plant height (PH), root length (RL), number of leaves (NL), stem diameter (SD), root dry weight (RDW), stem dry weight (SDW), leaf dry weight (LDW), leaf area (LA), shoot biomass (SB), total biomass (TB), biomass partitioning of root (PBr), biomass partitioning of stem (PBs), and biomass partitioning of leaf (PBl) (Figure 1). Most of the variables assessed (PH, RL, NL, RDW, SDW, LDW, SB, and TB) were statistically higher at 49 dae than at 28, 35, and 42 dae. This is logical since growth is irreversible and accumulative in the plant and is reflected in greater height, number of leave, s and total biomass. In contrast, SD at 35 and 42 dae (0.234167 and 0.231500 cm, respectively) were statistically equal and was larger at 28 and 49 dae (Figure 1).

In the case of partitioning root, stem, and leaf biomass, the proportions are determined by time; in the first sampling at 28 dae, the proportion of root is smaller and increases gradually until the last sampling at 49 dae, when there is a larger proportion of roots. The variables PBs and PBl are the opposite case: a larger proportion of leaves and stems is found in the first samplings and decreases gradually until the proportion is smaller in the 49 dae sampling (Figure 1).

The growth indexes relative growth rate (RGR) and net assimilation rate (NAR) were not significantly different in the samplings. In contrast, leaf area ratio (LAR), leaf weight ratio (LWR), and specific leaf area (SLA) at 35 dae were statistically superior with 56.747 cm^2^ day^−1^plant^−1^, 0.082789 g g^−1^, and 2028.06 cm^2^ g^−1^plant^−1^, respectively, indicating that the plantlet leaves were thinner than at 42 and 49 dae due to the high SLA value. The lowest values were 41.26 cm^2^ day^−1^plant^−1^, 0.061688 g g^−1^, and 935.92 cm^2^ g^−1^plant^−1^ for LAR, LWR, and SLA, respectively, at 42 dae (Table 2).

### 2.3. Growth and Development of Stevia Plantlets in Different Months of the Year

Significant differences were found in rooted plantlet (RP), PH, RL, NL, SD, RDW, SDW, LDW, LA, SB, TB, PBr, PBs, and PBl among the experiments set up on different dates (January through November). 

**Figure 2:**
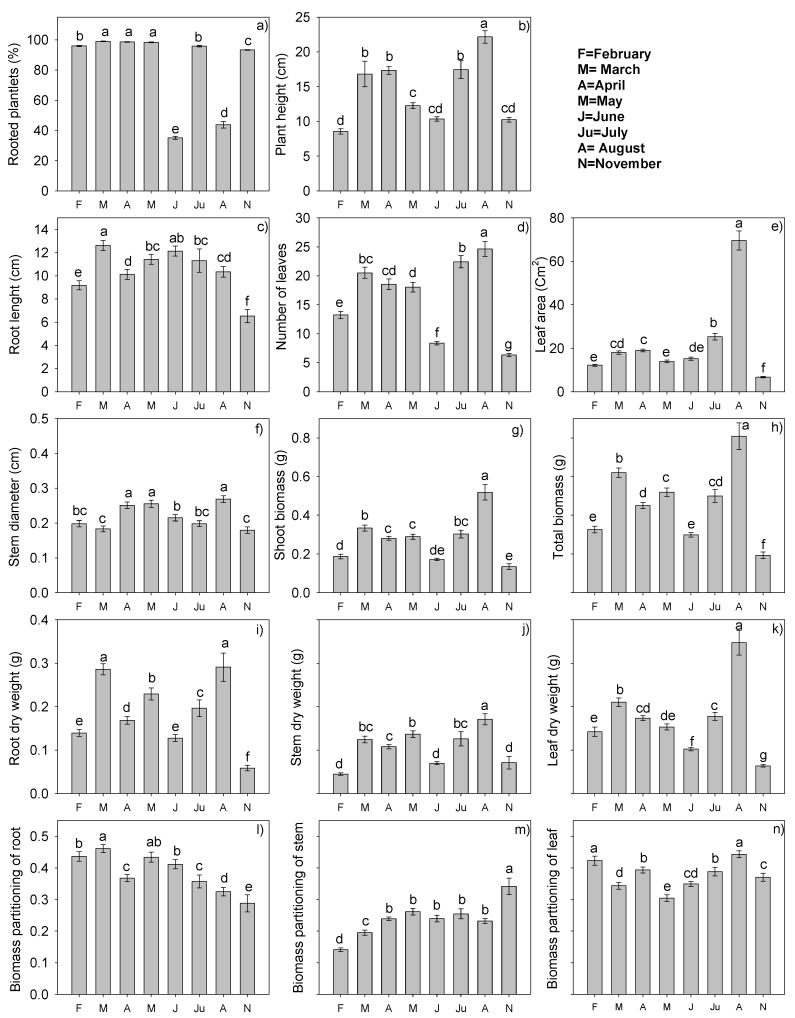
Average values of (**a**) rooted plantlet (RP, %), (**b**) plant height (PH, cm), (**c**) root length (RL, cm), (**d**) number of leaves (NL), (**e**) leaf area (LA, cm^2^), (**f**) stem diameter (SD, cm), (**g**,**h**) shoot and total biomass (SB and TB, g), (**i**,**j**,**k**) root, stem leaf dry weight (RDW, SDW, LDW, (**g**), and (**l**,**m**,**n**) biomass partition of root, stem, and leaf (PBr, PBs, PBl) in stevia plantlets established in different months of the year. Each value is the average of 60 data (5 replications of each of the 3 treatments in each of the 4 samplings) Means with the same letter in each figure are not significantly different (Tukey, *p* < 0.05).

The percentage of rooted plantlets is the main variable to consider in an asexual propagation system. In the March, April and May experiments, the percentages were statistically equal and higher than those of other months, with rooting percentages of 98.25%–98.96 %. In July (95.79%) and February (95.87%), rooting percentages were statistically equal. The experiment set up in November produced 93.17% of rooted plantlets. In contrast, June and August had the lowest percentages, 35.08% and 43.67%, respectively. For this reason, planting in these months is not economically feasible. In terms of plant height, plants in the August experiment were statistically superior, with 22.16 cm, followed by those in the March, April, and July experiments, which were statistically equal with values of 16.81, 17.33, and 17.44 cm, respectively. Stevia planted in February had the lowest PH. Roots of March plants were significantly longer (12.6 cm) than those of plants established in the other months, followed by June plants with 12.13 cm. In contrast, plants established in November had the shortest RL, 6.5 cm (Figure 2).

The number of leaves in the experiments oscillated from 6.37 for plants of the November experiment to 24.65 leaves/plant in the August experiment. The number of leaves in the August experiment was the largest, followed by that of plants of the experiments established in July, March, and April with 22.43, 20.52, and 18.55 leaves/plant, respectively. Leaf area of the plants of the August experiment was also the statistically highest with 69.59 cm^2^, followed by those of the July and April experiments with 25.3 and 18.97 cm^2^, respectively. Plants of the experiments established in November had the lowest value, 6.66 cm^2^ (Figure 2). Stem diameter of plants established in April, May, and August was statistically equal, 0.251, 0.255, and 0.269 cm, respectively, and larger than those of other months, followed by those of the June experiment with 0.215, February with 0.200, and July with 0.198 cm. In contrast, plants established in March and November had the smallest SD (Figure 2). 

Root dry weights of the experiments set up in March and August were significantly the highest values, 0.2858 and 0.2908 g plant^−1^, followed by plants established in May and July with 0.2291 and 0.1963 g plant^−1^. Root dry weights of those established in February and June were statistically equal, and plants established in November had the lowest result in root dry weight, 0.0586 g plant^−1^ (Figure 2). Stem dry weight of plants established in August was the highest, 0.1711 g plant^−1^, followed by those of May with 0.1367 g plant^−1^; March and July were statistically equal with 0.1244 and 0.1258 g plant^−1^, respectively. The lowest SDW values were found for plants established in February, June, and November, which were statistically equal. For leaf dry weight, the August experiment plants were statistically superior with 0.3470 g plant^−1^. In contrast, the November experiment resulted in the lowest LDW, 0.06378 g plant^−1^ (Figure 2).

Shoot biomass in the August experiment was statistically superior, with 0.5181 g plant^−1^, followed by that of the March and July experiments, 0.3345 and 0.3032 g plant^−1^, respectively. SB of the April and May experiments was statistically equal and lower than those mentioned above. The experiment with the lowest value was November with 0.1349 g plant^−1^. Total biomass followed the same trend as shoot biomass. The August experiment resulted in the highest value, 0.8089 g plant^−1^, followed by that of March with 0.6205 g plant^−1^, while the lowest value was for the November experiment with 0.1935 g plant^−1^ (Figure 2).

Calculation of the biomass partition coefficients of the organs that make up the plant is an estimation of the plant’s development. The coefficients express a percentage of dry mass production in each organ: leaf, stem, root, flower, and fruit. For the partition of root biomass (PBr), the March experiment was statistically superior, with 0.4614, followed by May with 0.4341 and February and June, which were statistically equal with values of 0.4292 and 0.4116, respectively. The PBr of the April and July experiments were statistically equal and lower than those mentioned, with values of 0.3676 and 0.3573, respectively. The August and November experiments had the lowest values, with 0.3247 and 0.2879, respectively (Figure 2). The partition of stem biomass (PBs) of the November experiment had the highest value, 0.3418, followed by those of April, May, June, July, and August, which were statistically equal. In contrast, the February experiment had the lowest PBs values (Figure 2). The leaf biomass partitions of the August and February experiments, with values of 0.4432 and 0.4239, respectively, were statistically equal and higher than those of the other experiments. The April and July experiments followed with statistically equal values. The lowest PBl was that of the May experiment with 0.3042 (Figure 2). 

Net assimilation rates were not statistically different among the different experiments. In contrast, there were significant differences for RGR, LAR, LWR, and SLA. The variable RGR was statistically the highest in the August experiment, with 0.0773 g cm^-2^ day^−1^plant^−1^; the rest of the experiments had statistically equal values (Table 2). The leaf area ratio estimates the proportion of leaf area in which photosynthesis maintains the entire plant. In plants established in August, LAR was statistically superior, with 101.752 cm^2^ day^−1^plant^−1^, followed by 54.60, 48.28, and 43.20 cm^2^ day^−1^plant^−1^ corresponding to the July, June, and April experiments, respectively. Those of March and May experiments were statistically equal and the lowest of all the experiments (Table 2). 

The leaf weight ratio estimates the plant’s leafiness. Again, the August experiment resulted in the statistically highest value, 0.105242 g g^−1^, followed by those of February, April, and July, which were statistically equal. May had the lowest LWR value. Specific leaf area estimates leaf thickness, and higher values indicate thinner leaves. The August experiment again resulted in the highest value 5374.1 cm^2^ g^−1^plant^−1^; therefore, the leaves are very thin. In contrast, the March and May experiments resulted in smaller values, 450.7 and 378.4 cm^2^ g^−1^plant^−1^, respectively, indicating thicker leaves (Table 2).

It is important to note that, although the August experiment resulted in many statistically superior variables, the most important variables, such as rooted plantlets and root length, had very low values, as well as very thin leaves. For these reasons, propagation in this month is not favorable. This is possibly related to the high temperatures that occur during this month: it was the only month in which daily low temperatures were above 17 °C (Table 1). 

### 2.4. Effect of hormones on Growth and Development of Stevia Plantlets

**Figure 3:**
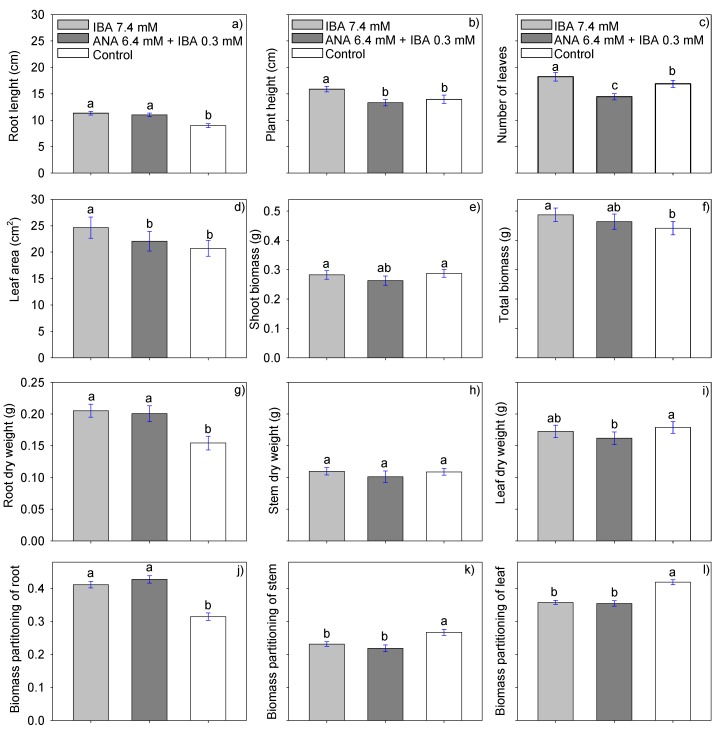
(**a**) Root length, (**b**) plant height, (**c**) number of leaves, (**d**) leaf area, (**e**) shoot biomass, (**f**) total biomass, (**g**) root dry weight, (h) stem dry weight, (**i**) leaf dry weight, and (**j**,**k**,**l**) biomass partition of root, stem, and leaf of stevia plantlets treated with growth regulators IBA 7.4 mM, ANA 6.4 mM + IBA 0.3 mM, and control. Each value is the average of 160 data (5 plantlets of each of the three treatments in each of the 4 samplings of each of the 8 experiments) Means with the same letter in each figure are not significantly different (Tukey, *p* < 0.05).

Significant differences in RP, PH, RL, NL, RDW, LDW, LA, SB, TB, LWR, LAR, and SLA were found among the evaluated treatments. However, there were no significant differences in SD and SDW, RGR, and NAR (Figure 3, Table 2). 

We found that the hormones indol-3 butyric acid (IBA) 7.4 mM and alphanaphthylacetamide (ANA) 6.4 mM + IBA 0.3 mM produce the same effect, which was statistically superior to the control, on plantlet rooting (83.03% and 82.5%), root length (11.33 and 11.01 cm), root dry weight (0.2051 and 0.2005 g), proportion of root biomass (0.4111 and 0.4272). It should be mentioned that the control obtained 81.7% of rooted plantlets. Moreover, IBA 7.4 mM was statistically superior to ANA 6.4 mM + IBA 0.3 mM and the control in plant height (15.88 cm), number of leaves (18.24), leaf area (24.64 cm^2^), total biomass (0.4875 g), and specific leaf area (1625.73 cm^2^ g^−1^plant^−1^). Also, IBA at 7.4 mM was statistically superior to ANA 6.4 mM + IBA 0.3 mM and equal to the control in SB (0.2876 and 0.2876) and LAR (49.86 and 47.29). In contrast, the control was statistically superior in LDW, PBs, PBl, and LWR, with values of 0.17876 g, 0.2670, 0.4188, 0.0872 g g^−1^, respectively. These results clearly show that when hormones are applied, the cutting prioritizes root production. It is important that the plantlet has enough roots to supply nutrients in later stages of development. In contrast, the control without hormones produced leaves.

## 3. Discussion

### 3.1. Temperatures during Development of the Experiments

Britos and Park (2016) have stated that appropriate temperatures for stevia propagation are between 18 and 33 °C [19]. However, daily highs were registered above 33 °C from February to November, and likewise, daily lows in all months were below 18 °C, indicating that the temperature was not the most adequate. Nevertheless, the percentages of rooted plantlets were high: 95.79%–98.96% in the February, March, April, May, and July experiments and 93.17% in the November experiment. The experiments established in June and August had extremely low percentages of rooted plantlets, 35.08 and 43.67%, respectively. Therefore, in these three months, propagation is not feasible [20]. 

### 3.2. Growth and Development of Stevia Plantlets at 28, 35, 42 and 49 Dae

Because plant growth and development are accumulative, total biomass was small in the first samplings. By the fourth and last sampling, SB, TB, PH, RDW, SDW, and LDW were higher. Although there are few references on the coefficients of biomass distribution in stevia plantlets during the process of propagation, studies on this species report that initially biomass is prioritized in leaves and later in stem, reaching proportions of 3 to 1 [21]. This was also observed in our research on the first two sampling dates when more biomass was distributed in the leaves, followed by root and stem, but on the third and fourth sampling dates there was a larger proportion in the root and the smallest proportion in the stem (Figure 1); this is logical since propagation requires plantlets with a well-developed root system to support transplant.

### 3.3. Growth and Development of Stevia Plants in Different Months of the Year

Asexual propagation is one of the best techniques for propagating stevia cuttings [13]. Micropropagation can obtain 85% propagated plantlets, 60% by cuttings, and 25.51%–40% by seed. Thus, this last method is the least used for propagation [13]. Moreover, propagation by seed results in high phenotypic variability [19] and the method is made difficult by the very small seeds, which lose viability after only a few days in storage [13], limiting this type of propagation [12]. It is therefore not recommended for use in a production system. 

In our study, the percentage of propagated plants was excellent when established in five of the eight months tested with percentages of 96%–99%, and 93.17% in November. Although the August experiment resulted in statistically superior values in PH, NL, SD, LA, SB and TB, and in RDW, SDW, LDW, PBl, LAR, LWR, and SLA, the percentage of rooted plantlets was 44%. Therefore, August and also June (33%) are not favorable for stevia propagation under the conditions of this study; according to Herrera (2012), mortality should not surpass 5%. Other studies on in vitro propagation in controlled conditions (25 °C and 16 h photoperiod) report that they found a higher percentage of viability, 60%, of propagated cuttings in November and October and that establishment in April produced a larger quantity of biomass but low percentages of cutting viability [13]. Their results may be related to the conditions in which the mother plants were found. 

The number of leaves the cuttings have when they are propagated also affects plantlet rooting. With four pairs of leaves, rooting is poor, especially in February; with two pairs of leaves rooting is better in February, and with three pairs of leaves rooting is better in April [18]. For our study, two pairs of leaves were left on the cuttings that were propagated. However, in February, no outstanding results in RDW were found, like the findings of [18], indicating that climatic conditions in which propagation takes place affect the response of the propagated plants. In another study with *Ficus binnendijkii*, concentrations of 4000 and 6000 mg/L IBA and different planting dates, late June and early September, were tested. They reported that September was the appropriate time to propagate this species [16]. As in our experiment, propagation in June did not produce good results. 

Our study found that IBA 7.4 mM was the hormone that produced the best results. This treatment resulted in statistically higher values in PH, NL, LDW, LA, TB, LAR, and SLA than the control and equal to ANA 6.4 mM + IBA 0.3 mM in the variables RL, RDW, and PBr. It was also statistically superior to ANA 6.4 mM + IBA 0.3 mM, and equal to the control in SB. In this respect, Muñoz and Molina (2016) found that the rooting percentage of juvenile *Myrceugenia exsucca* cuttings increased from 66.7% (control) to 88.3% when they applied 5000 mg L−1 IBA. In contrast, Kassahum and Mekonnen (2011) found no differences in the number of leaves per stevia plant with hormone application. The same authors found significant differences in the survival rate; with an application of IBA, the 75.55% survival was statistically equal to the control (73.61%) and superior to ANA with 64.72%; they, therefore, suggest that application of hormones is not necessary. In contrast, in our study, the control was outstanding only in the variables LWR, PBs, and PBl. Likewise, Babaie et al. (2014) reported that the use of hormones such as IBA at dosages of 4000 and 6000 mg/L in *Ficus binnendijkii* produces longer roots, heavier root fresh weight, and higher survival rate than the control. López et al. (2016) also reported that IBA at 1.0 ppm produces higher plants and longer roots in stevia [14]. Moreover, other studies have reported that other hormones, such as IAA at a concentration of 0.25 mg/L or ANA at 0.25 or 0.50 mg/L, also help in the formation of stevia roots [22]. ANA has been reported to stimulate cell elongation, growth, division, and differentiation, as well as to regulate abscission and stimulate the growth of adventitious roots [23]. 

The place from which the cuttings are taken influences the number of leaves on the propagated plant and survival rate. Cuttings from the apical part produce 8.22 leaves, while those from the middle part produce 5.60 leaves. Apical cuttings also have a higher survival rate, 80.18%, while those from the middle part have a 62.4% survival rate [7]. In this study, the cuttings were obtained from the apical part of the plant and had an average of 18.23 leaves plant^−1^ on the four sampling dates.

## 4. Materials and Methods

The study was conducted in Ciudad Guzmán, Jalisco, Mexico. Eight experiments were set up (February, March, April, May, June, July, August, and November). Three treatments were tested in each experiment): T1, indol-3 butyric acid (IBA) 7.4 m; T2, alphanaphthylacetamide (ANA) 6.4 mM + IBA 0.3 mM; and T3, control. The experimental design was completely randomized with five replications; the experimental unit (each replication) was a tray with 50 Morita II stevia plants. Analysis of the results was 3 × 4 × 8 factorial, where one factor was the three root-promoting treatments, the other factor was the sampling date, and the other was establishment date. Peat moss and farm soil in a 1:1 proportion were used as substrate. 

Five plants from T1 (IBA 7.4 mM), T2 (ANA 6.4 mM + IBA 0.3 mM), and T3 (control) of each of the eight experiments were sampled on each sampling date (28, 35, 42, and 49 days after establishment (dae)). The variables evaluated were rooted plantlets (RP, %), root length (RL, cm) from the root neck to the root tip (to calculate this variable, 5 replications of each of 50 plantlets for each treatment evaluated 49 dae). Stem diameter (SD, cm) was measured with a Truper electronic vernier; plant height (PH, cm) from root neck to the plant apex, measured with a 30 cm ruler; number of leaves (NL), leaf area (LA, cm^2^), calculated using Image J. software on a photograph, for which the leaves were placed on a white plane surface and a measuring scale in cm. Dry weight of root (RDW, g), stem (SDW, g), and leaf (LDW, g) were determined after drying in a Binder® drying oven, series FD at 60 °C for 72 h [24]. Each dried organ was weighed on an analytical balance (Saitorius, Mod. T214S). With the data of dry weight, growth indexes were calculated: relative growth rate (RGR, g g^−1^ day^−1^ plant^−1^), calculated following Sedano et al. (2005) [25]; net assimilation rate (NAR, g cm^−1^ day^−1^ plant^−1^) calculated according to Aguilar et al. (2005) [26]; leaf area relation (LAR, cm^2^ g^−1^ plant^−1^) calculated as the ratio between total leaf area and total plant dry weight; leaf weight relation (LWR g g^−1^), determined as the quotient between dry weight of the leaf area overplant dry weight; specific leaf area (SLA cm^2^ g^−1^ plant^−1^), referring to leaf area per unit of leaf weight. In addition, development indexes were calculated: coefficients of biomass partition of each organ (CBPx), leaf (CBPl), root (CBPr), stem (CBPs) with the formula CBPx = dry weight of each organ (DWx)/dry weight of the entire plant. An analysis of variance was performed and means compared with Tukey (α = 0.05) in the Statistical Analysis System® (SAS) version 9.1 [27]. For the analysis, eight experiments with 4 samplings were considered. 

## 5. Conclusions

The most adequate months of establishment for stevia propagation are February to July, with the exception of June. Cuttings can be established in November, but the percentage of rooted plantlets in our study was 93.17%. Establishing stevia propagation in June or August is not recommended. 

The best growth hormone treatment was IBA at 7.4 mM, which produced statistically higher PH, NL, LDW, LA, TB, LAR, and SLA than the control. This treatment was equal to that with ANA at 6.4 mM + IBA 0.3 mM in the variables RL, RDW, and PBr, and statistically superior to ANA 6.4 mM + IAB 0.3 mM and equal to the control in the variable SB. The control plants produced more shoot biomass and developed fewer roots.

## Figures and Tables

**Table 1:** Daily high, low, and average temperatures (°C) registered in the greenhouse located in Ciudad Guzmán, Jalisco, Mexico, from January to December 2017.

Month												
Temperature	Jan. (°C)	Feb. (°C)	Mar. (°C)	Apr. (°C)	May (°C)	June (°C)	July (°C)	Aug. (°C)	Sep. (°C)	Oct. (°C)	Nov. (°C)	Dec. (°C)
Low	5.8	7.3	8.4	5.9	8.9	10.1	15.9	17.3	15.3	13.6	6.9	6.4
High	32.0	35.5	36.8	41.4	42.7	41.6	33.6	32.9	33.7	34.9	33.3	32.0
Average	16.4	19.2	20.2	22.1	24.9	22.9	22.5	22.5	21.8	21.6	18.7	16.6

**Table 2:** Relative growth rate (RGR, g cm^−2^ day^−1^plant^−1^), net assimilation rate (NAR, g cm^−2^ day^−1^plant^−1^), leaf area ratio (LAR, cm^2^ day^−1^plant^−1^), leaf weight ratio (LWR, g g^−1^), and specific leaf area (SLA, cm^2^ g^−1^plant^−1^) of stevia plantlets with different establishment dates and growth regulators.

	RGR	NAR	LAR	LWR	SLA
date	**Sampling**				
35	0.036106^a^	0.0009481^a^	56.747^a^	0.082789^a^	2028.06^a^
42	0.032940^a^	0.0007141^a^	46.482^b^	0.069684^b^	1317.48^b^
49	0.024810^a^	0.0006367^a^	41.263^c^	0.061688^c^	935.92^c^
**Month**					
February	0.03020^b^	0.0009348^a^	41.202^de^	0.082318^b^	838.2^d^
March	0.02123^b^	0.0008937^a^	29.759^f^	0.058670^d^	450.7^e^
April	0.01741^b^	0.0004562^a^	43.201^d^	0.075211^b^	923.5^cd^
May	0.01017^b^	0.0007527^a^	27.350^f^	0.045195^e^	378.4^e^
June	0.02334^b^	0.0005088^a^	48.278^c^	0.061167^cd^	1160.2^c^
July	0.03716^b^	0.0008458^a^	54.599^b^	0.075678^b^	1513.8^b^
August	0.07730^a^	0.0007989^a^	101.752^a^	0.105242^a^	5374.1^a^
November	0.03348^b^	0.0009396^a^	39.173^e^	0.067615^c^	778.3^d^
**mM**		**Hormone**			
IBA 7.4	0.028792^a^	0.0007577^a^	49.859^a^	0.064068^b^	1625.73^a^
ANA 6.4 + IBA 0.3	0.034672^a^	0.0007955^a^	47.347^b^	0.062876^b^	1354.38^b^
Control	0.030391^a^	0.0007457^a^	47.287^b^	0.087218^a^	1301.34^b^

Means with the same letter in column are not significantly different (Tukey, *p* < 0.05).
